# Adherence to dietary recommendations according to the General Dietary Behavior Inventory (GDBI) and its association with bioelectrical impedance analysis (BIA) parameters among young, healthy and normal weight women

**DOI:** 10.1186/s40795-026-01260-0

**Published:** 2026-01-29

**Authors:** Nadja Knoll-Pientka, Dorina Schils, Katrin Mantwill, Hannah Dinse, Eva-Maria Skoda, Alexander Bäuerle, Martin Teufel, Lars Libuda

**Affiliations:** 1 Clinic for Psychosomatic Medicine and Psychotherapy, University of Duisburg-Essen, LVR-University Hospital Essen, Essen, Germany; 2 Center for Translational Neuro- and Behavioral Sciences (C-TNBS), University of Duisburg-Essen, Essen, Germany; 3 Faculty of Food and Nutrition Science, Hochschule Niederrhein, University of Applied Sciences, Mönchengladbach, Germany; 4 Faculty of Natural Sciences, Institute of Nutrition, Consumption and Health, Paderborn University, Paderborn, Germany

**Keywords:** Anorexia nervosa, Body composition, EDE-Q, DOS, Dietary behavior, GDBI

## Abstract

**Background:**

The General Dietary Behavior Inventory (GDBI) is a low effort instrument with only 16 items to assess the general dietary behavior based on the dietary recommendations of the World Health Organization and the German Nutrition Society. In an online survey with a convenience sample, higher total GDBI scores indicating healthier dietary behavior were associated with a lower body mass index (BMI).

**Aim:**

Since mean value of the self-reported BMI in that sample was in the overweight range, the aim of the current study was to examine the adherence to dietary recommendations using the GDBI in a sample of young and healthy women at the lower normal BMI range.

**Methods:**

In total, 63 women aged 22.2 ± 2.2 years with a mean BMI of 20.4 ± 1.0 kg/m^2^ were included in this study. Body composition was determined using bioelectrical impedance analysis (BIA). GDBI sum score and single item scores were compared with those of the validation study. Spearman correlations were calculated between the GDBI sum score and age, BMI, waist circumference (WC), and BIA parameters.

**Results:**

The mean GDBI score of 55.76 ± 6.46 in this sample was similar to that of the validation study. Regarding single items, the most pronounced difference compared with the validation study was found for the scores of item 2 indicating lower consumption of animal products in the current study. However, this item was not concordant with all further items. The GDBI sum score correlated with age, but neither with BMI nor WC nor any BIA parameter.

**Conclusion:**

In conclusion, other than expected we did not find a higher GDBI score compared with that of the validation study. Moreover, in this homogenous, healthy sample of young women at the lower normal BMI range, healthier dietary behavior as indicated by higher GDBI scores does not explain differences in BMI or body composition.

**Trial registration:**

The study is registered at the German Clinical Trials Register (DRKS-ID: DRKS00030472). Date of registration: October 10th, 2022, retrospectively registered.

**Supplementary Information:**

The online version contains supplementary material available at 10.1186/s40795-026-01260-0.

## Introduction

Individual dietary behavior is an important resource for maintaining mental and physical health and can be preventive for non-communicable diseases, such as diabetes, heart disease and stroke [1–5]. Therefore, assessing dietary behavior is of great importance for target-oriented dietary interventions. Here, not only mere food, nutrient or energy intake should be considered, but also behavioral aspects, since, for instance, self-preparation of food and how it is eaten were shown to be associated with a favorable weight development or diet quality [6, 7]. For this reason, the General Dietary Behavior Inventory (GDBI) was developed and validated in a large convenience sample [8]. The GDBI is based on the recommendations for a healthy and wholesome diet of the German Nutrition Society (version of 2017–2024) [9–11] and the World Health Organization [12, 13]. It is a low effort tool focusing on general dietary behavior by addressing both food intake as well as the above-mentioned behavioral aspects using 16 items with a 5-point bipolar scale [8]. As an example for food intake, “I eat at least 2 servings of fruit daily.” (= behavior A, 5 points) is contrasted with “I never eat fruit.” (= behavior B, 1 point); as example, for how food is eaten “When taking my meals, I like to take my time.” (= behavior A, 5 points) is contrasted to “I like to take my meals quickly.” (= behavior B, 1 point). The scores of each item are summed up to calculate the GDBI sum score, which ranges from 16 to 80. In the validation study in a convenience sample of 428 participants (mean BMI: 26.8 ± 7.8 kg/m^2^), mean GDBI sum score was 56.7 ± 7.9 [8]. Moreover, the GDBI sum score was negatively associated with BMI, indicating that a dietary behavior less in accordance with current dietary recommendations was associated with a higher BMI, and positively associated with self-reported physical and mental health as well as life satisfaction [8].

In addition, the GDBI was applied in a cross-sectional study of 458 people affected by obesity with a mean BMI of 43.2 ± 8.3 kg/m^2^ [14]. In line with the finding of a negative relationship with BMI found in the convenience sample, the mean GDBI sum score of that sample was 54.4 ± 7.8 [14], and hence, slightly lower compared with that of the convenience sample [8]. However, within that sample there was even a positive association with BMI and no relationship with any further health related outcomes, deeming the GDBI not to be suitable among people affected by severe obesity [14].

In the present study, we aimed at investigating the adherence to dietary recommendations measured by the GDBI in a sample of young, healthy, and normal weight women with a BMI of 18.5 to 22.0 kg/m^2^. The rationale for investigating dietary behavior among young, healthy women within this lower normal BMI range might not be their urgent need for nutritional assessment or counselling per se, but they can be considered a good reference sample and aim for an effective therapy for patients with eating disorders such as bulimia nervosa or anorexia nervosa. Regarding the negative association between BMI and GDBI score in the convenience sample [8] and the lower GDBI sum score in the sample of people affected by obesity [14], we hypothesized that this sample with a BMI in the lower normal range should have a higher GDBI sum score compared to the convenience sample. Moreover, we aimed at investigating whether parameters of body composition assessed by bioelectrical impedance analysis (BIA) are associated with the GDBI score.

## Methods

This investigation was part of an intervention study exploring the influence of water consumption immediately before BIA measurements on impedance raw data and derived body compartments, which took place at the Clinic for Psychosomatic Medicine and Psychotherapy of the LVR-University Hospital Essen, Germany, from September to December 2021 [15]. For that study, each participant was asked to come to the study center in the morning in a fasting state on two consecutive days. For the present investigation, we only used the data of the baseline BIA measurement of the first study day. Only for one participant, the baseline measurement of the second study day had to be used, as this participant did not come in a fasting state to the study center on the first day.

### Participants

Participants were recruited through public notices and various communication channels of social networks (e.g. Facebook, LVR-website, Instagram). Participants had to fulfill the following inclusion criteria: 18 to 25 years, female, Caucasian origin, and a BMI from 18.5 to 22.0 kg/m^2^. Exclusion criteria were the presence of diseases or conditions that make it difficult to come to the study center in a fasting state, diseases that affect osmoregulation (e.g., diabetes mellitus, kidney failure/renal insufficiency, heart insufficiency or anorexia nervosa), the use of medications, especially those that affect osmoregulation (e.g., such as diuretics or corticosteroids), the presence of a stent or implanted electronic devices, the presence of a current or past eating disorder diagnoses, lack of adequate German language skills, the presence of pregnancy or lactation, as well as the presence of prostheses or amputated body parts. To ensure adequacy, participants were screened in a brief telephone interview. One both study days, participants had to abstain from food for at least eight hours before measurement. Likewise, neither drinking alcohol, nor visiting the sauna nor performing any exhausting exercise was allowed the day before each study day to leave osmoregulation in a natural state. According to these criteria, 149 interested candidates were screened, 79 fulfilled inclusion criteria and finally, 63 were included in the study, as 16 did not appear to the study center due to several reasons such as illness or time reasons.

### Anthropometrics and BIA

Body height was determined with an accuracy of ± 0.5 cm using a stadiometer (seca GmbH & Co. KG, Hamburg). Waist circumference (WC) was measured midway between the lowest rib and the uppermost boarder of the iliac crest with a non-stretchable circumference measuring tape (seca 201) with an accuracy of ± 0.5 cm.

BIA measurements were conducted in standing position in light clothing with the medical Body Composition Analyzer 515 (mBCA515; seca GmbH & Co. KG, Hamburg) using an eight-electrode technique with each two electrodes on both sides at the handle and the platform, a current of 100 µA and frequencies of 5 and 50 kHz. Each BIA measurement had a duration of 17 s. Before BIA measurements, participants were asked to be seated for 15 min.

This device was validated against respective gold standard reference methods in study samples with a BMI range of 18.5–35 kg/m^2^ [16, 17]. Parameters of body composition (absolute fat mass (FM), relative fat mass (%FM), fat free mass (FFM), skeletal muscle mass (SMM), total body water (TBW), extracellular water (ECW), and visceral adipose tissue (VAT)) were derived from internal proprietary equations which consider the measured raw values resistance R and reactance Xc [16, 17].

Body mass was also measured with the mBCA515. Indices of body mass, FM, FFM and SMM were calculated by dividing the respective value (in kg) by height (in m) squared. These indices, and TBW, ECW, ECW/TBW as well as the phase angle (PhA), a measure for body cell tissue quantity and quality which is calculated from the directly measured R and Xc [18], were used for further analyses. Although WC is a good estimate of VAT [19], we found a low and even negative correlation between these two parameters among our study participants (Spearman ρ = − 0.16, *p* = .212). Based on this finding and the fact that estimated VAT by mBCA515 only explains 81% of VAT measured by magnetic resonance tomography [17], we decided not to consider VAT in all further analyses.

BIA data were exported from the medical PC software seca analytics 115, version 1.4.1040.6789.

### Assessment instruments

After baseline BIA measurement, participants completed the GDBI [8] as well as two questionnaires pertaining to pathological eating behavior symptomatology, the German version of the Eating-Disorder Examination Questionnaire (EDE-Q) [20] and the Düsseldorf Orthorexia Scale (DOS) [21].

As described above, the GDBI measures general dietary behavior and contains 16 items with a 5-point bipolar scale. Details on single items are shown in Table 2. As an example, “I eat at least 2 servings of fruit daily.” (= behavior A, 5 points) is contrasted with “I never eat fruit.” (= behavior B, 1 point). The scores of each item are summed up to calculate the GDBI sum score, which ranges from 16 to 80, with higher sum scores indicating dietary behavior more in accordance with current dietary recommendations. Cronbach´s α of this scale was 0.73 [8].

The EDE-Q is a 36-item self-report measure, which spans a 28-day period and scores with a seven-point Likert scale [20, 22]. It measures severity of eating-disorder psychopathology with four subscales (eating concern; weight concern; shape concern; and dietary restraint). Cronbach’s α and retest reliability of the German version were 0.97 and 0.88, respectively [20].

The DOS consists of 10 items capturing orthorexic eating behavior referring to the last seven days on a four-point Likert scale. High scores indicate high levels of orthorexic eating behavior. Cronbach’s α and retest reliability were 0.84 and 0.79, respectively [21].

Both the EDE-Q and DOS were utilized to ensure that participants do not show pathological eating behaviors.

### Statistics

All statistical analyses were performed using IBM SPSS Statistics^®^ version 24 [23]. Means and standard deviations were calculated for descriptive statistics. The deviation (in %) of each GDBI item of the present investigation from the respective item of the validation study [8] was calculated as follows: ((item x [present investigation] – item x [validation study])/ item x [validation study])*100. Correlation analysis of the GDBI sum score with age, BMI, WC and BIA parameter with and without adjusting for age were performed using Spearman ρ. Since dietary behavior reflects very different components which do not necessarily have to correlate with each other, Cronbach’s α is not applicable for the GDBI [8]. Hence, only inter-item correlations of all 16 GDBI items were considered. As item 2 was negatively correlated with all further items, a modified score in which this item was omitted was also correlated with BMI, WC and BIA parameter, and again, with and without adjusting for age. For all analyses, the level of significance was set at *p* < .05. Due to the exploratory character of this study corrections for multiple testing were not performed.

### Ethical consideration

The study was approved by the Ethics Committee of the Medical Faculty of the University of Duisburg-Essen (20-9525_2-BO). Examinations were conducted in accordance with the Declaration of Helsinki. All participants gave written informed consent to the study conditions before participating in the study.

## Results

### Sample characteristics

The participants of the sample were 22.2 ± 2.2 years old and had a BMI of 20.4 ± 1.0 kg/m^2^. Relative fat mass was 23.8 ± 3.7% (Table 1). Global EDE-Q and DOS scores were 0.3 ± 0.5 and 14.9 ± 3.5, respectively, and thus, indicate that neither eating disorder psychopathology [20] nor orthorexic eating behavior [21] were pronounced among our study participants.

**Table 1:** Descriptive statistics of age and anthropometric data and their correlation with GDBI sum score

Item	Mean	SD	Spearman correlation coefficient	*P*
Age [years]	22.2	2.2	− 0.279	**0.027**
Body Mass Index (BMI) [kg/m^2^]	20.4	1.0	0.028	0.827
Waist Circumference (WC) [m]	0.64	0.03	− 0.055	0.667
Fat-Free Mass Index (FFMI) [kg/m^2^]	15.5	0.9	0.166	0.193
Skeletal Muscle Mass Index (SSMI) [kg/m^2^]	7.1	0.6	0.111	0.388
Total Body Water (TBW) [kg]	33.0	3.4	0.170	0.183
Extracellular Water (ECW) [kg]	14.0	1.4	0.229	0.071
ECW by TBW [%]	42.5	1.4	0.062	0.630
Fat Mass Index (FMI) [kg/m^2^]	4.9	0.9	− 0.141	0.269
Relative Fat Mass (%FM) [%]	23.8	3.7	− 0.171	0.179
Phase Angle (PhA) [°]	5.2	0.5	0.042	0.746

*N* = 63

### Descriptive statistics and correlations of the GDBI

**Fig. 1:**
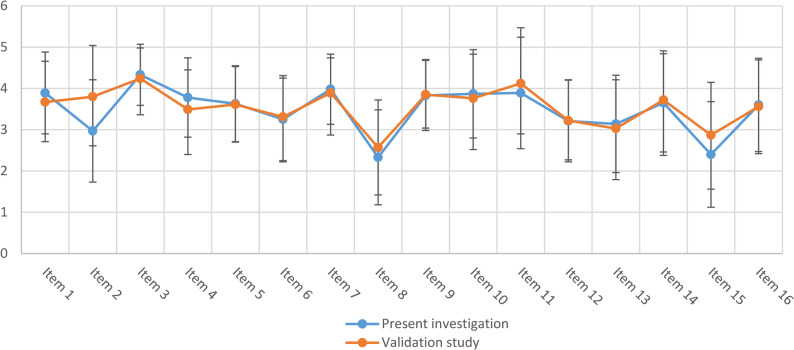
Per item comparison of mean value of GDBI items between present investigation and validation study [8] All items except 12 & 14 are coded 5 (first mentioned behavior) to 1 (second mentioned behavior) with a higher score indicating dietary behavior more in accordance with current recommendations; mean values of items 12 & 14 are recoded

Since the GDBI sum score theoretically ranges from 16 (= being least in adherence with dietary recommendations) to 80 points (= being most in adherence with dietary recommendations), the observed average value of 55.76 ± 6.46 in the present investigation was slightly above the midpoint of this scale, and – contrary to our hypothesis – even slightly lower compared to that of the validation study (56.7 ± 7.9 [8]). Single items ranged between 2.33 ± 1.15 for item 8 (i.e., consumptions of sweets) and 4.33 ± 0.74 for item 3 (i.e., inclusion of plant-based products). Average scores of single items deviated by -1.7 ± 8.0% from the respective scores of the validation study. Highest deviations were found for item 2 (i.e., consumption of animal products) and item 15 (i.e., eating under distraction) with − 21.8% and − 16.4%, respectively (Fig. 1; Table 2).

**Table 2:** Scores of general dietary behavior inventory (GBDI) items and deviations from the validation study

Item^1^	Mean (inverted items 12 & 14 recoded)	SD	Deviation from respective item score of validation study [8] in %
GDBI – 1 “I eat different foods every day” vs. “I eat the same foods every day”	3.89	0.99	6.0
GBDI – 2 “My meals always include animal products (e.g., meat, fish, eggs, dairy products such as yogurt, cream, cheese)” vs. “My meals do not include animal products (e.g., meat, fish, eggs, dairy products such as yogurt, cream, cheese)”	2.97	1.24	-21.8
GBDI – 3 “My meals always include plant-based products (e.g., vegetables, grain products, tofu products)” vs. “My meals do not include plant-based products (e.g., vegetables, grain products, tofu products)”	4.33	0.74	2.1
GBDI – 4 “I eat at least 2 servings of fruit daily.” vs. “I never eat fruit.”	3.78	0.96	8.3
GBDI – 5 “I eat at least 3 servings of vegetables daily.” vs. “I never eat vegetables.”	3.63	0.92	0.6
GBDI – 6 “I eat whole grain products (e.g., whole grain pasta, whole grain bread) instead of white flour products (e.g., “conventional” pasta, white bread/grain bread) every day.” vs. “I eat white flour products (e.g., “conventional” pasta, white bread/grain bread) instead of whole grain products (e.g., whole grain pasta, whole grain bread) every day.”	3.25	1.00	-1.8
GBDI – 7 “I always consume vegetable fats (e.g., margarine and oils based on canola, olive, sunflower) instead of animal fats (butter, lard, etc.).” vs. “I always consume animal fats (butter, lard, etc.) instead of vegetable fats (e.g., margarine and oils based on olives, canola, sunflowers).”	3.98	0.85	2.3
GBDI – 8 “I do not eat sweets (e.g., chocolate, cookies, pastries).” vs. “I eat sweets (e.g., chocolate, cookies, pastries) every day.”	2.33	1.15	-9.3
GBDI – 9 “I do not eat fast food and convenience foods (e.g., frozen pizza, microwave ready-to-eat meals).” vs. “I eat fast food and convenience foods (e.g., frozen pizza, microwave ready-to-eat meals) daily.”	3.83	0.85	-0.5
GBDI – 10 “I do not drink sugary/sweetened beverages (e.g., juices, soda, sweetened coffee/tea).” vs. “I drink sugary/sweetened beverages (e.g., juices, lemonade, sweetened coffee/tea) daily.”	3.87	1.07	2.9
GBDI – 11 “I consume at least 1.5 L of fluid daily (excluding alcoholic beverages or beverages containing sugar/sweeteners).” vs. “I consume less than 1.5 L of fluid (excluding alcoholic beverages or beverages containing sugar/sweeteners) daily.”	3.89	1.35	-5.6
GBDI – 12 “When preparing meals, it is important to me that they are prepared quickly (e.g., quick frying, quick/spicy sautéing).” vs. “When preparing meals, it is important to me that they are prepared slowly (e.g., slow cooking/frying with medium heat).”	3.21	0.99	-0.3
GBDI – 13 “When seasoning my meals, I make sure to use as little salt as possible.” vs. “I like to use a lot of salt when seasoning my food.”	3.14	1.18	3.6
GBDI – 14 “I drink alcohol regularly (daily or even every weekend).” vs. “I don’t drink alcohol or drink it very rarely (only on special occasions).”	3.65	1.19	-1.9
GBDI – 15 “I prefer to eat in peace without being distracted by anything.” vs. “While eating, I like to occupy myself with other topics (e.g., watching TV, thinking about work, reading).”	2.40	1.28	-16.4
GBDI – 16 “When taking my meals, I like to take my time.” vs. “I like to take my meals quickly.”	3.60	1.13	1.1
GDBI – Sum score	55.76	6.46	-1.7

*N* = 63; ^1^all items except 12 & 14 are coded 5 (first mentioned behavior) to 1 (second mentioned behavior) with a higher score indicating dietary behavior more in accordance with current recommendations; mean values of items 12 & 14 are recoded

Item 2, which deviated most from that of the validation study, was negatively correlated with all further items (Table S1), and with a corrected GDBI sum score in which item 2 was omitted (*r* = − .371, *p* < .05, Table S2).

While the GDBI sum score correlated negatively with age (*r* = − .279, *p* = .027, Table 1), no significant correlations were observed with BMI, WC or any of the BIA parameters (Table 1). Adjusting for age did not lead to any further significant association (Table S3). In the correlation analysis with a GDBI score in which item 2 was omitted, the negative association with age persisted. Moreover, it resulted in one further significant correlation with ECW (*r* = .259, *p* = .04), which however, when adjusted for age, did not persist (*r* = .198, *p* = .123, Table S3).

## Discussion

In the present study, we applied the GDBI [8] for the first time in a homogenous sample of young, healthy women at the lower normal BMI range. In contrast to our hypothesis, we found a slightly lower GDBI sum score indicating a somewhat lower adherence to dietary recommendations from the German Nutrition Society [9–11] and the World Health Organization [12, 13] compared with that of the validation study [8]. Moreover, contrary to the validation study using a convenience sample with heterogenous BMI, we did not find an association with BMI or any BIA derived parameter.

Considering the negative association of the GDBI sum core with BMI in the convenience sample [8], we expected to find a higher GDBI sum score in the current study, since mean BMI of the participants in our study was substantially lower compared with that of the validation study (20.4 kg/m^2^ vs. 26.8 kg/m^2^). Interestingly, the sum score was even slightly lower among our participants (55.8 vs. 56.7 [8]). Several aspects should be considered to explain this somewhat unexpected finding. First, body weight regulation is a complex interaction of several factors such as genetic factors [24] including variation in individual energy expenditure [25], and physical activity [26] rather than exclusively dietary behavior. Second, the dietary recommendations on which the GDBI is based aim at general health-promoting aspects and not only at body weight regulation. In fact, total energy intake as the dietary parameter which should be most directly related to body weight is not considered in the GDBI. Third, as resting energy expenditure is higher among younger adults (< 25 years) compared with adults aged 25 years and older, total energy expenditure might be higher by at least 100 kcal even at constant physical activity levels [27]. Hence, even if the similar GDBI sum scores indicate similar energy intakes in both samples, it might have different effects on body weight between the 18-to-25-year-olds of our study compared with the participants the validation study at an average age of 34.8 years [8].

However, on item level, our investigation also revealed two GDBI item scores to be substantially lower compared with those of the convenience sample in the validation study. The largest discrepancy was found for item 2 (2.97 vs. 3.80 [8]), , which decreased internal consistency most pronouncedly in our sample. This item assesses whether animal products are always part of the meals (highest score) vs. not being part of the meals (lowest score). In the validation study of the English version of the GDBI (GDBI-E), two clusters of dietary behavior were found, and participants following dietary behavior more in accordance with dietary recommendations scored higher in all single items except for item 2 compared with those following a less healthy dietary behavior [28]. In that study, vegans were overrepresented with 29% of study participants. Per definition, vegans leave out animal products and item 2 of the GDBI is scored lowest, when animal products not being part of meals [8], which could explain the lower scoring of item 2 in that sample [28]. Indeed, in Germany, highest percentages of vegetarians and vegans were found among under 30-years-olds and women [29]. Moreover, daily consumption of vegetarian or vegan alternatives for animal products was highest in that age class with 17% compared to 8% in the general German population [30]. As our sample only consists of women with a mean age of 22.2 years, vegetarians/vegans and/or daily consumption of alternatives for animals products might have been more prevalent in the current study compared to the sample of the German GDBI validation study consisting of both men and women with an average age above 30 years [8]. Accordingly, participants of our study might have tended more often towards the lower scoring behavior (i.e. animal product not being part of the meals) compared with the validation study of the GDBI [8].

Nevertheless, following a vegan diet might be a healthy dietary alternative, in case animal products are replaced adequately, which is not addressed in the current version of the GDBI. Moreover, whether the inclusion of animal products to each meal can be considered more in accordance with current recommendations clearly depends on the amount and kind of animal products consumed in a day, as for instance, meat and sausages should not exceed 300 to 600 g per week according to the recommendations of the German Nutrition Society (version of 2017) [9]. In addition, the recommendations of the German Nutrition Society were renewed in 2024 [31]. In this regard, the amount of animal products was reduced to 300 g/week for meat and sausages, and instead of three portions of milk and dairy products per day, still only two are recommended [31]. Hence, including animal products in each meal would most properly exceed these renewed recommendations. Furthermore, we found that only item 2 negatively correlated with each other item as well as with the total GBDI score in our study, which confirms findings from a previous study [28]. Therefore, a revision of the GDBI pertaining to this item seems reasonable, ideally with separate evaluations of meat (products) and milk (products). Concurrently, adequate plant-based alternatives should be addressed in a revised GDBI version.

Furthermore, our sample scored significantly lower regarding item 15 (2.40 vs. 2.87 in the convenience sample [8]), which assesses whether one prefers eating without any distraction (highest score) vs. eating while being occupied with something as e.g. watching TV (lowest score). One explanation for this finding might be the potentially different living conditions and/or marital status of the participants. In the convenience sample, most of the participants were married or in a partnership. By contrast, the young women of our study, who most likely might have been students due to the recruitment process, might less often share common family meals. Other distractions such as for instance smartphone use might replace the common table talks.

Although the participants of our investigation were within a narrow age range of 18 to 25 years, we found a negative association between age and the GDBI sum score. This association was not found in the validation study despite a clearly broader age-range [8]. The age range applied in our investigation is specific, as early adulthood is a phase in which individuals become increasingly autonomous from their parents by for instance moving out, and this process might yet have been more fulfilled among the older participants of our study sample. Hence, the older our participants were, the lower might have been their parents’ influence and support regarding food supply which in turn might have led to dietary behavior less in accordance with dietary recommendation.

Pertaining to BMI, WC and BIA parameters, we did not find any association of the GBDI sum score. Taking the aforementioned considerations regarding the revision of the GBDI into account, we conducted a sensitivity analysis. When omitting item 2 for the GBDI score, no significant associations were observed in age-adjusted analysis (Table S3). Compared with the respective validation study of both GDBI [8] and GDBI-E [28], both in which a lower BMI was associated with a dietary behavior more in accordance with currant recommendations, the participants of the present investigation had a clearly narrower BMI range (20.4 ± 1.0 kg/m^2^ vs. 26.8 ± 7.8 kg/m^2^ [8] & 24.8 ± 5.9 kg/m^2^ [28]). The low variance of this variable could have made it difficult to find an association with BMI or parameters of body composition. Moreover, in samples with presence of individuals with overweight or moderate obesity as in both validation studies [8, 28], a healthy eating behavior might be preventive against further increase of body weight, but might not have additional effects for individuals in the lower normal weight range. Neither is it desirable nor intention of healthy or wholesome dietary recommendations to further reduce BMI or particular body compartments, when being already in the (lower) normal range as found among the participants of our study [32].

### Limitations

As to the inclusion criteria, which were chosen to obtain a potential reference sample and aim for an effective therapy for patients with eating disorders such as bulimia nervosa or anorexia nervosa, the sample was rather homogenous regarding BMI range, sex, and age. While this homogeneity has the advantage that large effects of confounding factors should be avoided, our results cannot be generalized to ages, sexes and weight classes. Moreover, healthiness was based on self-assessment and focused on the absence of any current or past eating disorder as well as any disorder affecting osmoregulation. Consequently, we cannot exclude apparent health issues beyond these aspects. Another limitation is that due to the small sample size, our statistical power might be inappropriate to detect weak correlations between GDBI and e.g., single BIA parameters. Additionally, data were derived from a cross-sectional study. For multicausal outcomes such as the body weight status and body composition with large interindividual differences, a longitudinal design analyzing the associations between individual changes in eating behavior and concomitant changes in these outcomes might be more appropriate. Moreover, as to the exploratory character of our study, we did not correct for multiple testing. Accordingly, type I error risk may be increased which is why the only significant relation, i.e., between age and GDBI sum score, might have been falsely positive. Hence, confirmation of this correlation in independent samples is required.

## Conclusion

Using the GDBI in a sample of healthy, young women with a BMI in the lower normal weight range, we found – contrary to our expectation – a slightly lower GDBI sum score compared with that of the validation sample with substantially higher mean BMI. While the current study indicates that an improvement in GDBI score and, thus, eating behavior, does not seem to further improve body weight status and body composition in an already healthy sample, longitudinal studies with larger sample sizes are needed to examine its long-term relevance.

## Supplementary Information

Supplementary Material 1.

## Data Availability

Raw data are available from the corresponding author on reasonable request.

## Abbreviations

BIA: Bioelectrical impedance analysis
BMI: Body mass index
ECW: Extracellular water
FFM: Fat free mass
FM: Fat mass
GDBI: German Dietary Behavior Inventory
GDBI-E: English version of GDBI
PhA: Phase angle
R: Resistance
SMM: Skeletal muscle mass
TBW: Total body water
WC: Waist circumference
Xc: Reactance
Z: Impedance
