# Supplementary material for: Adherence to dietary recommendations according to the General Dietary Behavior Inventory (GDBI) and its association with bioelectrical impedance analysis (BIA) parameters among young, healthy and normal weight women
Source: BMC Nutr. 2026 Jan 29;12:42. doi: 10.1186/s40795-026-01260-0 (PMC12924241; doi:10.1186/s40795-026-01260-0)
Supplement: Supplementary file 1 — Supplementary Material 1. [file 40795_2026_1260_MOESM1_ESM.docx]

**Supplementary data**

**Table S1 Inter-item correlations of the GDBI**

| GDBI – 1 | | GDBI - 2 | GDBI - 3 | GDBI - 4 | GDBI - 5 | GDBI - 6 | GDBI - 7 | GDBI - 8 | GDBI - 9 | GDBI - 10 | GDBI - 11 | GDBI - 12 | GDBI - 13 | GDBI - 14 | GDBI - 15 | GDBI - 16 |
| --- | --- | --- | --- | --- | --- | --- | --- | --- | --- | --- | --- | --- | --- | --- | --- | --- |
| GDBI - 1 |  |  |  |  |  |  |  |  |  |  |  |  |  |  |  |  |
| GDBI - 2 | -.227 |  |  |  |  |  |  |  |  |  |  |  |  |  |  |  |
| GDBI - 3 | **.339**** | **-.321*** |  |  |  |  |  |  |  |  |  |  |  |  |  |  |
| GDBI - 4 | **.349**** | -.060 | .265* |  |  |  |  |  |  |  |  |  |  |  |  |  |
| GDBI - 5 | **.541**** | -.165 | .725 | **.418**** |  |  |  |  |  |  |  |  |  |  |  |  |
| GDBI - 6 | .176 | -.162 | .211 | .279* | .067 |  |  |  |  |  |  |  |  |  |  |  |
| GDBI - 7 | .171 | **-.335**** | **.392**** | **.253*** | **.404**** | .156 |  |  |  |  |  |  |  |  |  |  |
| GDBI - 8 | .033 | **-.252*** | .057 | .068 | .071 | .080 | .104 |  |  |  |  |  |  |  |  |  |
| GDBI - 9 | .092 | -.188 | **.349**** | .169 | **.267*** | -.042 | -.070 | .143 |  |  |  |  |  |  |  |  |
| GDBI - 10 | .109 | -.015 | .034 | .177 | .149 | -.030 | -.055 | **.336**** | **.523**** |  |  |  |  |  |  |  |
| GDBI - 11 | -.131 | **-.282*** | .151 | .181 | .084 | **.309*** | .153 | -.028 | **.278*** | .057 |  |  |  |  |  |  |
| GDBI - 12 | **.356**** | -.087 | **.501**** | .152 | **.422**** | .240 | .062 | .066 | **.370**** | **.254*** | .066 |  |  |  |  |  |
| GDBI - 13 | .028 | -.228 | .019 | -.029 | -.026 | .147 | -.014 | .024 | -.039 | .079 | -.071 | .169 |  |  |  |  |
| GDBI - 14 | .172 | -.008 | .152 | .086 | .088 | **.373**** | -.006 | .145 | -.029 | -.010 | .006 | .158 | .128 |  |  |  |
| GDBI - 15 | .151 | -.033 | -.040 | .152 | -.012 | .034 | -.157 | .018 | .109 | .215 | .129 | .177 | -.038 | .018 |  |  |
| GDBI - 16 | **.264*** | -.147 | -.032 | .215 | .138 | .162 | .178 | .029 | .161 | **.251*** | .109 | .176 | .056 | .015 | .223 |  |

GDBI, general dietary behavior inventory; significant correlations are bold, *, p<0.05, **, p<0.01

**Table S2 Change of Cronbach’s α per item omission**

|  | **Correlation coefficient between respective item and a corrected GDBI sum score (of which the respective item is omitted)** | **Cronbach’s α if the respective item is omitted** |
| --- | --- | --- |
| GDBI - 1 | .375 | .562 |
| GDBI - 2 | -.371 | .687 |
| GDBI - 3 | .428 | .564 |
| GDBI - 4 | .444 | .552 |
| GDBI - 5 | .484 | .548 |
| GDBI - 6 | .349 | .566 |
| GDBI - 7 | .169 | .593 |
| GDBI - 8 | .133 | .601 |
| GDBI - 9 | .347 | .570 |
| GDBI - 10 | .357 | .563 |
| GDBI - 11 | .141 | .603 |
| GDBI - 12 | .509 | .541 |
| GDBI - 13 | .025 | .620 |
| GDBI - 14 | .211 | .588 |
| GDBI - 15 | .169 | .597 |
| GDBI - 16 | .303 | .571 |

GDBI, general dietary behavior inventory; Cronbach’s α of GDBI in present investigation: **.601**

| **Item** | **Mean** | **SD** | **Spearman ρ** | ***P*** | **Spearman ρ_adjusted for age** | ***P*** | **Spearman ρ_omitting item 2** | ***P*** | **Spearman ρ_omitting item 2_adjusted for age** | ***P*** |
| --- | --- | --- | --- | --- | --- | --- | --- | --- | --- | --- |
| **Age [years]** | 22.2 | 2.2 | -.279 | **.027** |  |  | -.269 | **.033** |  |  |
| **Body Mass Index [kg/m^2^]** | 20.4 | 1.0 | .028 | .827 | -.031 | .812 | .032 | .802 | -.024 | .852 |
| **Waist Circumference [m]** | 0.64 | 0.03 | -.055 | .667 | -.096 | .458 | -.031 | .810 | -.069 | .595 |
| **Fat-Free Mass Index [kg/m^2^]** | 15.5 | 0.9 | .166 | .193 | .102 | .432 | .176 | .168 | .115 | .375 |
| **Skeletal Muscle Mass Index [kg/m^2^]** | 7.1 | 0.6 | .111 | .388 | .036 | .782 | .123 | .337 | .052 | .687 |
| **Total Body Water value (TBW) [kg]** | 33.0 | 3.4 | .170 | .183 | .082 | .525 | .199 | .119 | .118 | .363 |
| **Extracellular Water value (ECW) [kg]** | 14.0 | 1.4 | .229 | .071 | .163 | .206 | .259 | **.040** | .198 | .123 |
| **ECW by TBW value [%]** | 42.5 | 1.4 | .062 | .630 | .087 | .499 | .075 | .557 | .100 | .438 |
| **Fat Mass Index [kg/m^2^]** | 4.9 | 0.9 | -.141 | .269 | -.136 | .293 | -.139 | .276 | -.134 | .300 |
| **Relative Fat Mass [%]** | 23.8 | 3.7 | -.171 | .179 | -.143 | .266 | -.174 | .173 | -.147 | .255 |
| **Phase Angle [°]** | 5.2 | 0.5 | .042 | .746 | .026 | .839 | .035 | .786 | .020 | .878 |

**Table S3 Correlations of anthropometrics with GDBI sum score and a GDBI score of which item 2 was omitted (with and without adjusting for age)**
